# Effect of Physical Therapy with Combined Resistance Exercises and Vigorous Walking in Older Adult Women with Chronic Non-Specific Pain: A Randomized Controlled Trial

**DOI:** 10.3390/life16020341

**Published:** 2026-02-16

**Authors:** Rocío Cogollos-de-la-Peña, Gemma Victoria Espí-López, Anna Arnal-Gómez, Lucas Monzani, Juan J. Carrasco, Laura Fuentes-Aparicio

**Affiliations:** 1 Faculty of Health Science, Universidad Europea de Valencia, Pg. de l’Albereda, 7, El Pla del Real, 46010 Valencia, Spain; rocioinmaculada.cogollos@universidadeuropea.es; 2 Department of Physiotherapy, Faculty of Physiotherapy, University of Valencia, Gascó Oliag St., 5, 46010 Valencia, Spain; gemma.espi@uv.es (G.V.E.-L.); juan.j.carrasco@uv.es (J.J.C.); laura.fuentes@uv.es (L.F.-A.); 3 Exercise Intervention for Health Research Group (EXINH-RG), Department of Physiotherapy, University of Valencia, 46010 Valencia, Spain; 4 Physiotherapy in Motion. Multispeciality Research Group (PTinMOTION), Department of Physiotherapy, University of Valencia, Gascó Oliag St. 5, 46010 Valencia, Spain; 5 Ivey Business School at Western University, 1255 Western Rd., London, ON N6G 0N1, Canada; lmonzani@ivey.ca

**Keywords:** older adult women, nonspecific chronic pain, elastic band, vigorous walking

## Abstract

Background: Age-related hormonal changes in older women accelerate bone and muscle loss, leading to postural dysfunction and chronic musculoskeletal pain. This study aimed to evaluate the short-term effects of a physical therapy program combining elastic band exercises and vigorous walking on pain, thoracic mobility, and functional capacity in older adult women. Methods: A multicenter randomized controlled trial was conducted older adult women (60–80 years) with chronic non-specific musculoskeletal pain, allocated to an elastic band plus vigorous walking group (EBWG), a vigorous walking group (VWG), or a control group (CG). A total of 91 participants completed all of the assessments. Outcomes included pressure pain threshold (PPT), self-reported pain (VAS), thoracic mobility (UPC, LWC), functional capacity (5XSTS), and perceived improvement (PGIC), evaluated at baseline, after a 4-week intervention, and at 4-week follow-up. Results: The EBWG demonstrated greater improvements in PPT (+0.66 kg/cm^2^ at T2), upper chest expansion (+1.00 cm), and 5XSTS performance (−1.7 s) compared to the control group. The VWG showed significant reductions in overall pain (−0.9 points) and lumbar pain (−1.7 points). Improvements in PPT and thoracic mobility in the EBWG exceeded MDC/MCID thresholds, indicating clinically meaningful changes. Vigorous walking alone improved self-reported pain but was less effective than the multicomponent program. Conclusions: A 4-week combined program of elastic band exercises and vigorous walking produced clinically relevant improvements in pain threshold, thoracic mobility, functional capacity, and perceived change compared to walking alone or usual activity. These findings support the clinical utility of short, feasible, multicomponent interventions for managing chronic musculoskeletal pain in older women.

## 1. Introduction

Aging is a universal biological process that affects all body systems, with the musculoskeletal system being particularly vulnerable [1]. In older adult women, hormonal changes associated with menopause accelerate the loss of bone and muscle mass, increasing the risk of sarcopenia and osteoporosis [2]. These physiological changes, combined with joint degeneration, often result in postural alterations and chronic musculoskeletal pain, especially in the thoracic region and upper limbs [3].

The thoracic region plays a central role in posture, breathing, and balance. Chronic pain in this area is associated with reduced muscle activation efficiency and altered movement patterns, which may further restrict thoracic mobility [4]. Thoracic kyphosis is more prevalent in older adult women, who tend to exhibit increased kyphosis and lumbar lordosis angles compared to men [5], which can impair respiratory mechanics and reduce ventilatory efficiency [6].

Physical therapy is a fundamental tool to counteract these functional declines. Resistance exercises using elastic bands have shown positive effects on muscle strength, postural control, and pain reduction in older adults [7]. When targeting the upper limbs, these exercises help improve scapular stability and proprioception, enhancing postural alignment during daily activities [8].

In parallel, vigorous walking is a well-established aerobic activity with benefits in delaying functional decline. It contributes to improved stability, balance, coordination, and endurance in older adults [9], while also being effective in reducing chronic musculoskeletal pain [10]. Furthermore, physical interventions have shown significant effectiveness in managing chronic pain where pharmacological approaches fall short [11].

Therefore, the hypothesis of our study was that an intervention combining elastic band exercises with vigorous walking could significantly reduce non-specific chronic musculoskeletal pain in older adult women over 4 weeks, while improving thoracic mobility, functional capacity, and perceived physical condition.

The objective of this study was to evaluate whether a short-term (4-week) combined program of elastic band exercises and vigorous walking, compared to vigorous walking alone, improved thoracic mobility, reduced pain, and produced perceived physical improvement in physically active older adult women with sessions performed twice a week for 40 min.

## 2. Materials and Methods

### 2.1. Study Design

A multicenter, randomized, double-blind clinical trial was carried out between October 2022 and November 2023. The research was conducted at the Laboratories of the Faculty of Physical Therapy of the University of Valencia (Spain) and the Faculty of Physical Education and Sport, Charles University (Czech Republic). The potential bias arising from two different locations was controlled by the stay of some co-authors in the collaborating country. This exchange facilitated the transfer of the methodology for both the evaluation and execution of techniques uniformly, and monthly follow-up checks were conducted. Although no quantitative inter-site reliability coefficients were recorded, methodological consistency was ensured through the standardized training of evaluators, shared operational manuals, monthly supervision meetings, reciprocal observation of assessment and intervention sessions, and additional oversight conducted by the research teams through cross-country exchanges during evaluation and intervention procedures. No protocol deviations were documented during the monitoring period. Participants were randomly allocated to one of three groups: the vigorous walking group (VWG), the elastic band and vigorous walking group (EBWG) or the control group (CG). Evaluations of musculoskeletal pain and functional capacity were performed at three different time points: baseline (T0), immediately following the 4-week intervention period (T1), and after a subsequent 4-week follow-up phase (T2). Prior to inclusion, all participants received complete information about the study procedures and provided written informed consent. The study was conducted in accordance with the Declaration of Helsinki, and the protocol was reviewed and approved by the Human Research Ethics Committee of the University of Valencia (reference number: 1393203). Furthermore, the study is registered on ClinicalTrials.gov under the broader project with identifier NCT04345211. The Czech part of the experiment was approved by the UK FTVS Ethics Committee (266/2022). All methodological procedures adhered to the standards and recommendations outlined in the CONSORT guidelines for reporting randomized controlled trials.

### 2.2. Participants

The sample consisted of older adult women between 60 and 80 years of age who experienced persistent non-specific musculoskeletal or osteoarticular pain for more than three months without a diagnosed pathology, who did not present any diagnosed cardiovascular or respiratory conditions, and who were allowed to continue pre-existing analgesic medication and physiotherapy that had been stable for at least six months prior to study entry, but were not permitted to initiate new medications or other therapeutic interventions during the study period. In addition, they walked regularly at least two times a week. Participants were excluded if they met any of the following criteria: current tobacco use; reliance on assistive devices for ambulation; presence of acute musculoskeletal or osteoarticular pain at the time of evaluation; identifiable pathologies as the primary cause of the pain; or cognitive impairment, defined as a score below 25 on the Mini-Mental State Examination (MMSE).

### 2.3. Outcomes

The treatment and assessments were performed by two separate physiotherapists, each with over 15 years of experience: one administered the treatment exclusively, while the other, who was blinded to group assignment, conducted the evaluations independently. Prior to the evaluation, all participants received a detailed explanation of the procedures. An assessment, including that of the sociodemographic, anthropometric and clinical information, was conducted, followed by the study assessments.

The sociodemographic and clinical data were recorded, including marital status, educational level and employment status as well as age, sex, body weight measured with a Tanita BC 601 (TANITA Ltd., Tokyo, Japan) and height measured with a SECA 213 stadiometer (Seca Ltd., Hamburg, Germany). Subsequently, body mass index (BMI) (kg/m^2^) was calculated. In addition, information was collected on medical history, including hypertension and cholesterol levels. The Spanish version of the abbreviated Charlson Comorbidity Index was used to classify comorbidity levels (0–1 points: no comorbidity, 2 points: low comorbidity, and ≥3 points: high comorbidity).

#### 2.3.1. Primary Outcome

##### Musculoskeletal Pain

Perceived pain was measured by the visual analog scale (VAS). The VAS measures pain intensity in adults using a 10 cm line indicating the extremes “no pain”, and “worst pain imaginable”. Pain below 4 cm is considered mild, between 4 and 7 cm it is considered to be of moderate intensity, and above 7 cm it is considered severe [12]. Non-specific perceived pain was assessed in the following areas: the head and neck (VAS-HN), shoulders (VAS-S), dorsal area (VAS-D), low-back area (VAS-L), and pelvic and hip area (VAS-PH); the overall self-reported pain was calculated (VASTOT). This scale is validated and reliable and the intraclass correlation coefficient (ICC) is 0.97 [13]. The minimal clinically important difference (MCID) for VAS (0–100 mm) in chronic non-specific pain is ≈20–25 mm [14].

Pain pressure threshold (PPT) was measured by the minimal pressure (kg/cm^2^) which induces pain measured by pressure algometry (Wagner Instruments FDK 20, Wagner Instruments, Greenwich, CT, USA). The patient is seated, and the trapezius muscles are assessed bilaterally, performing three measurements in each muscle, with a period of thirty seconds rest between them. The average of the three scores was obtained for analysis, so we would consider the ICC = 0.76, the minimal detectable change (MDC) as 0.48–1 kg/cm^2^, and the MCID as between 0.51 and 2.24 kg/cm^2^ [15].

#### 2.3.2. Secondary Outcomes

To assess thoracic mobility, chest expansion was measured in centimeters using a flexible tape measure at two different levels of the rib cage. The measurement procedure consisted of determining the difference in thoracic circumference between maximal inhalation and maximal exhalation. For evaluating upper chest expansion (UPC), anatomical landmarks included the spinous process of the fifth thoracic vertebra, the midpoint of the clavicular line, and the level of the third intercostal space. In the case of lower chest expansion (LWC), the spinous process of the tenth thoracic vertebra and the xiphoid process served as reference points. Measurements were performed twice at each level, and the mean value of the two trials was used for analysis. Lower values were interpreted as indicative of reduced thoracic mobility and limited chest wall expansion [16]. The ICC was 0.90–0.93 for the upper portion and 0.85–0.86 for the lower portion and MCD should be above 3.60 for the upper and 4.40 for the lower [16].

Functional capacity was assessed using the five times sit-to-stand test (5XSTS). This test is employed to identify the risk of recurrent falls [17], with a cutoff time of more than 15 s indicating reduced functional capacity and a higher risk of falling. To perform the 5XSTS, participants were instructed to stand up and sit down five times as quickly as possible from a slightly padded, armless chair with a seat height of 43 cm. During the test, participants kept their arms crossed over their chest, stood up fully during each repetition, and made firm contact with the chair upon sitting. Timing started on the verbal cue “go” and ended when the participant sat down after the fifth repetition. A practice trial consisting of two repetitions was allowed before the timed tests. Two full test trials of five repetitions were then conducted, and the fastest time recorded was used for the final analysis (ICC ≈ 0.988–0.995) [18]. The MDC is approximately 2.5 s, while the MCID has been estimated to be around 2 to 3 s [19].

Perceived change following treatment: The patient’s subjective evaluation of the therapeutic effect was assessed using the patient global impression of change (PGIC) scale. This instrument captures the individual’s perception of health status change following the intervention. It includes a 7-point ordinal scale, where a score of 1 indicates they feel much worse and a score of 7 represents a feeling of being much better [20].

### 2.4. Interventions

The VWG conducted vigorous walking sessions on an outdoor circular track of approximately 400 m. Each session lasted 40 min and was carried out as a group, including all of the study participants, twice a week for 4 weeks under the supervision of a physiotherapist. The sessions began with a 10 min warm-up at a walking pace chosen by the participants, followed by a gradual increase in intensity until they reached a level where maintaining a conversation was no longer possible [21] or achieved a minimum score of 16 (“somewhat difficult”) on the Borg perceived exertion scale [22]. This scale was explained beforehand to ensure that each participant could adjust the intensity to match their physical capabilities. By completing both weekly sessions, participants met the WHO recommendations for vigorous aerobic physical activity (more than 75 min of vigorous aerobic exercise per week).

The EBWG first attended group session of self-administered protocol using elastic bands (EB), targeting the upper body, torso and lower extremities. The upper body and torso exercises aim to improve posture by promoting an upright posture and chest expansion. Lower limb exercises, which are simple and common among older people, contribute to pelvic alignment and indirectly improve torso posture and chest position. These exercises are designed to avoid overloading the upper limbs. Each exercise was performed five consecutive times at a controlled pace and always ensuring a slow return to the starting position, thus avoiding an abrupt recoil of the elastic band (see Supplementary Material File S1). Participants were instructed to reach a perceived exertion level of 16 (“somewhat difficult”) on the RPE (Rate of Perceived Exertion) scale, according to Fielding et al. [22], when applying external resistance using the elastic bands. This resistance was progressively increased when participants reached the desired level of exertion, using bands with higher tension. Green and red Akrafit elastic bands (https://www.herycor.com/, accesed on 25 July 2025) were used for the intervention, offering a resistance of between 13 and 20 N (green) and between 18 and 27 N (red) when stretched to twice their original length. After completing these exercises, participants engaged in a vigorous walking session, identical to that performed by the VWG, lasting 40 min at a high intensity. Walking was performed after the EB intervention, as this has been shown to enhance the synergistic effect of combining physical therapy and walking [23]. Adherence to the sessions was high, as all participants attended the scheduled sessions and consistently reached the target RPE of 16 during both the elastic band and walking components, and progression was individualized by adjusting the band tension or walking pace according to each participant’s performance. The actual dose of the intervention corresponded to two supervised sessions per week for the VWG, each lasting 40 min, during which participants consistently achieved the prescribed vigorous intensity (RPE 16). For the EBWG, it corresponded to two complete sessions of the protocol with elastic bands (five controlled repetitions per exercise), with individualized progression by increasing the resistance of the band, followed by 40 min of vigorous walking, as with the VWG, so that its approximate duration was 60 min.

The CG maintained their regular physical activity, which should include walking two days a week, but were not given any further instructions.

### 2.5. Sample Size Calculation

The sample size was calculated using G*Power 3.1.9.7. An “a priori” power analysis was performed for three independent groups to detect moderate effect sizes (f (V) = 0.25; Cohen’s d = 0.50) with α = 0.05 and 1− = 0.80. A Cohen’s d value of d = 0.50 was chosen based on prior studies, which showed that non-pharmaceutical interventions tend to have moderate-to-small effects on pain reports, as measured by the VAS [24]. The results indicated a minimum sample size of 98 participants was needed to achieve sufficient statistical power. We estimate a 15% loss, so we will need a total of 112 participants as the sample size required for our study.

### 2.6. Randomization and Blinding

A simple randomization process was performed. An independent researcher, not involved in participant recruitment, assessment, or intervention, generated the random allocation sequence. The initial sample was randomly assigned into three groups using sequentially numbered, sealed, opaque envelopes containing the group allocation. Allocation concealment was ensured as the envelopes were prepared in advance and opened only after baseline assessment. The study was double-blinded: the physical therapist who performed the assessments and the data analyst were unaware of group allocation. The physical therapist who delivered the intervention was blinded to the assessment results. Due to the nature of the intervention, participants could not be blinded, as they were aware of the group to which they were assigned.

### 2.7. Statistical Analysis

All statistical analyses were conducted using IBM SPSS Statistics 26 (IBM Corp., Armonk, NY, USA). Three multivariate mixed repeated-measures analysis of variance (RM-MANOVA) models and two mixed RM-ANOVA models were specified, one of which was a two-stage model (model E). This approach allows for the assessment of mean differences across groups (between-subject effects; VWG, EBWG and CG) and within-subject effects across sessions (T0: baseline, T1: post-intervention, T2: follow-up 4 weeks later) and their interaction effects (between–within effects) on the dependent variables.

Box’s test of the equality of covariance matrices (between-subjects) and Mauchly’s test of sphericity (within-subjects) were used. When the assumption of sphericity was violated, corrections based on the Epsilon statistics were applied (e.g., Greenhouse–Geisser or Huynh–Feldt).

Four models were constructed: model A assessed differences in the estimated mean pressure pain threshold (PPT) and differences in overall self-reported pain (VAS). Model B analyzed the differences in different body areas evaluated using the VAS (head–neck, shoulder, dorsal, lumbar and pelvis–hip). Model C assessed thoracic mobility; two different measures were analyzed for LWC and UPC. Model D examined changes in functional capacity using the five times sit-to-stand test (5XSST). Model E examined perceptions of change post-intervention (PGIC) and 4 weeks later. The results included F-values, statistical significance (*p*-values) and partial eta squared (η^2^_p_) as a measure of effect size. Effect size was interpreted as small (η^2^_p_ = 0.01, d = 0.2), medium (η^2^_p_ = 0.06, d = 0.5) and large (η^2^_p_ > 0.14, d > 0.8). When the main effects of the models indicated significant differences, post hoc tests were performed using the Bonferroni correction for multiple comparisons. The significance level was set to *p* < 0.05.

## 3. Results

**Figure 1:**
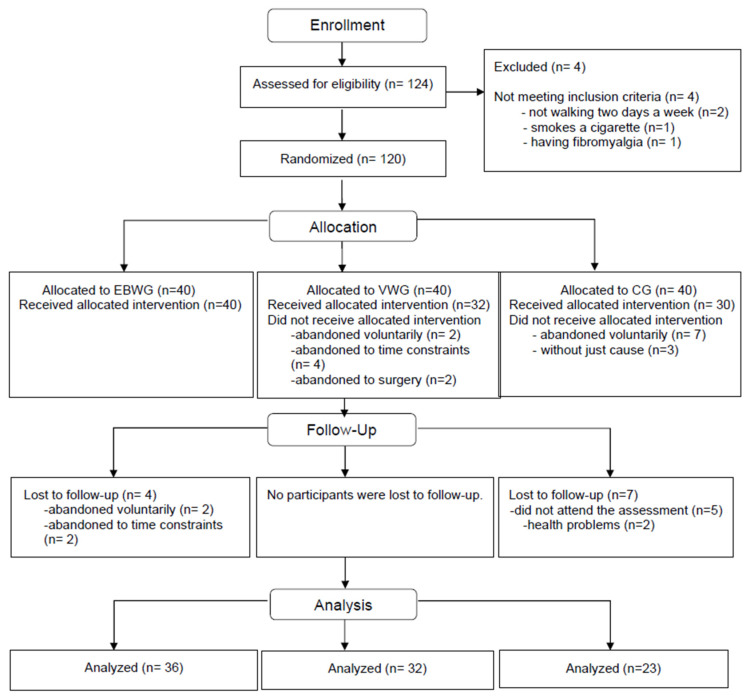
Flowchart according to CONSORT statement for the report of randomized trials.

From the 124 women who were interested in participating in the study, 120 met the inclusion criteria and were allocated, and 102 (40 in the EBWG, 32 in the VWG, and 30 in the CG) were finally analyzed (Figure 1).

The mean age of the participants was 70.01 ± 5.40 years, 56% were married, and 51% had a university degree. The mean weight was 68.02 kg, the mean height was 1.6 m, and the mean BMI was 26.64 ± 6.47 kg/m^2^. A total of 77.45% had no comorbidities, 73.53% were normotensive and only 40.20% had dyslipidemia. There were no statistical differences between the groups regarding their sociodemographic characteristics at baseline (Table 1).

Model A. Regarding the pain variables, first, the multivariate analysis indicated statistically significant main effects between subjects (F (4, 174) = 3.151; *p* = 0.016; η^2^_p_ = 0.068), within subjects over time (F (4, 85) = 3.85; *p* = 0.006; η^2^_p_ = 0.154), and a significant time-by-group interaction (F (8, 170) = 2.83; *p* = 0.006; η^2^_p_ = 0.118).

Follow-up univariate analyses showed significant differences in the variable overall VAS over time (F (1.796, 158.018) = 5.850; *p* = 0.005; η^2^_p_ = 0.062), and in time-by-group interaction in both overall VAS (F (3.591, 158.018) = 3.276; *p* = 0.016; η^2^_p_ = 0.069) and PPT (F (3.464, 152.400) = 3.497; *p* = 0.013; η^2^_p_ = 0.074). Moreover, PPT presented significant differences in the group factor (F (2, 88) = 6.592; *p* = 0.002; η^2^_p_ = 0.130).

Post hoc pairwise comparisons between groups for PPT revealed that significant differences between the EBWG and CG were observed at T1 (*p* = 0.020) and T2 (*p* = 0.004) with large effect sizes (d = 0.77 and d = 0.92). Also, baseline differences between the VWG and CG persisted throughout the study period (*p* < 0.05). Concerning the intra-group analysis, although the EBWG showed a trend towards improvement in overall VAS, the difference between T0 and T2 did not reach statistical significance (*p* = 0.074). The VWG experienced significant reductions in overall VAS scores between T0–T1 (*p* = 0.002) and T0–T2 (*p* < 0.001), reflecting a progressive improvement in self-reported pain. No significant changes in overall VAS were observed in the control group over time. For VAS outcomes, the reductions observed in the VWG between T0–T1 and T0–T2 exceeded the minimum clinically important difference reported for chronic non-specific pain (20–25 mm), supporting the clinical relevance of these findings. The EBWG showed a trend toward exceeding these thresholds, although changes did not consistently surpass the MCID.

**Figure 2:**
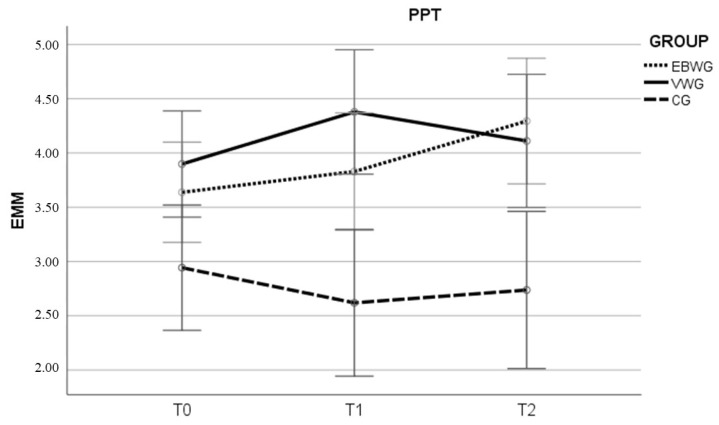
Estimated marginal means for pain pressure threshold. Note: EMM: Estimated marginal means; PPT: pain pressure threshold (kg/cm^2^); EBWG: elastic band and vigorous walking group; VWG: vigorous walking group; CG: control group; T0: baseline; T1: immediately following the 4-week intervention period; T2: after a subsequent 4-week follow-up phase.

For PPT, the EBWG demonstrated significant improvements in pain threshold between T0–T2 (*p* = 0.015, d = 0.40) and T1–T2 (*p* = 0.019, d = 0.26), indicating a sustained enhancement in pain tolerance over time, while the VWG showed a significant increase between T0 and T1 (*p* = 0.045) with a small effect size (d = 0.29). It also showed an MCD in the EBWG between T0–T2 and in the VGW group between T0–T1 (Table 2 and Figure 2). Additionally, the improvements observed in PPT in the EBWG between T0 and T2 exceeded the reported MDC range (0.48–1.0 kg/cm^2^) and fell within the MCID interval (0.51–2.24 kg/cm^2^), confirming that the change was not only statistically significant but also clinically relevant. In the VWG, the short-term improvement from T0 to T1 also surpassed the MDC threshold, indicating a clinically relevant increase in pain pressure tolerance.

Model B. Following the identification of significant differences in overall pain perception (VAS total), a more detailed analysis was conducted to explore variations in pain across specific anatomical regions. Multivariate analysis first revealed statistically significant within-subject differences over time (F (10, 79) = 2.693; *p* = 0.007, η^2^_p_ = 0.254).

Subsequent univariate analyses indicated significant effects of the time factor on lumbar pain (VAS lumbar) (F (1.990, 175.150) = 8.000; *p* < 0.001; η^2^_p_ = 0.083), as well as significant time-by-group interactions for neck and head pain (VAS neck and head) (F (3.680, 161.928) = 2.777; *p* = 0.033; η^2^_p_ = 0.059), and dorsal pain (VAS dorsal) (F (5.068, 159.780) = 2.830; *p* = 0.031; η^2^_p_ = 0.060).

Post hoc pairwise comparisons between groups did not reveal any statistically significant differences. However, within-group analyses showed that in the EBWG, lumbar pain showed a trend toward improvement, with near-significant reductions between T0–T1 (*p* = 0.069) and T0–T2 (*p* = 0.052), both associated with a medium effect size (d = 0.38). In contrast, the VWG exhibited significant reductions in cervical pain between T0 and T2 (*p* = 0.038, d = 0.33), and in dorsal pain between T0 and T1 (*p* = 0.022, d = 0.55). Lumbar pain in this group also improved significantly between T0–T1 (*p* = 0.004) and T0–T2 (*p* = 0.001), both with medium effect sizes (d = 0.58 and d = 0.64, respectively) (Table 2).

Model C. Multivariate analysis assessing thoracic mobility revealed statistically significant within-subject effects over time (F (4, 84) = 5.046; *p* = 0.001; η^2^_p_ = 0.194), as well as a significant time-by-group interaction (F (8, 168) = 2.258; *p* = 0.026; η^2^_p_ = 0.097).

Follow-up univariate analyses showed significant effects of time on upper chest expansion (UPC) (F (1.694, 147.408) = 13.804; *p* < 0.001; η^2^_p_ = 0.137) and lower waist circumference (LWC) (F (1,.823, 158.643) = 9.248; *p* < 0.001; η^2^_p_ = 0.096). Significant time-by-group interactions were found for LWC (F (3.647, 158.643) = 3.683; *p* = 0.009; η^2^_p_ = 0.078).

Post hoc pairwise comparisons between groups did not yield statistically significant differences. However, intra-group analyses revealed that the EBWG experienced significant improvements in UPC between T0–T1 (*p* < 0.001) and T0–T2 (*p* = 0.003), with medium effect sizes (d = 0.73 and d = 0.54, respectively). Significant improvements were also observed in LWC between T0–T1 (*p* < 0.001) and T0–T2 (*p* = 0.003), with medium and small effect sizes (d = 0.63 and d = 0.45, respectively), indicating enhanced upper thoracic expansion following the combined intervention. Importantly, the improvements in upper chest expansion (UPC) and lower chest expansion (LWC) observed in the EBWG exceeded the published MDC values (3.6 cm for UPC and 4.4 cm for LWC), indicating clinically relevant benefits in thoracic mobility beyond measurement error.

In the VWG, a near-significant increase in LWC was observed between T0 and T2 (*p* = 0.054) with a small effect size (d = 0.36) (Table 3).

Model D. A univariate analysis was conducted to assess functional performance using the five times sit-to-stand test (5XSTS). Results indicated statistically significant within-subject effects over time (F (1.726, 160.539) = 9.484; *p* <0.001; η^2^_p_ = 0.093) as well as a significant time-by-group interaction (F (3.452, 160.539) = 4.340; *p* = 0.004; η^2^_p_ = 0.085).

Post hoc pairwise comparisons between groups revealed a statistically significant difference between the EBWG and the CG at T0 (*p* = 0.020), with a small effect size (d = 0.34) which disappeared over time. Intra-group analysis showed that the EBWG experienced significant improvements in 5XSTS performance between T0–T1 and T0–T2 (*p* < 0.001), with medium effect sizes (d = 0.67 and d = 0.66, respectively), indicating a positive impact of the combined intervention on functional capacity. No significant changes were observed in the other groups (Table 3). Furthermore, the reductions in 5XSTS time observed in the EBWG between T0–T1 and T0–T2 surpassed both the MDC (~2.5 s) and the estimated MCID (2–3 s), confirming that the improvements reflect a clinically meaningful enhancement in functional capacity.

Model E. A univariate analysis was performed to assess the patients’ overall impression of change which was evaluated after 4 weeks of intervention (T1) and 4 weeks later (T2).

Results indicated statistically significant between-subject effects (F (2, 89) = 9.053; *p* < 0.001; η^2^_p_ = 0.169). Post hoc pairwise comparisons between groups revealed a statistically significant difference between the EBWG and the CG (*p* = 0.002) and between the VWG and the CG at T1 (*p* < 0.001), with both having a large effect size (d = 1.445 and d = 0.931, respectively). In the EBWG these differences are maintained over time (*p* = 0.032) with a medium-large effect size (d = 0.767). An intra-group analysis showed that VWG experienced significant improvements in their perception of change between T1–T2 (*p* = 0.008), with a small effect size (d = 0.435) (Table 3).

## 4. Discussion

This study demonstrates that the combination of elastic band exercises and vigorous walking (EBWG) provides complementary benefits over vigorous walking alone (VWG) and superior results compared to unstructured walking (CG) over four weeks. Improvements were observed in multiple clinical and functional dimensions. Specifically, the EBWG showed greater improvements than the VWG in pressure pain threshold (model A), thoracic mobility (UPC and LWC, model C), and functional capacity (5XSTS, model D), whereas reductions in self-reported pain were more prominent in the VWG. In the EBWG, greater pain tolerance was observed through significant increases in pressure pain threshold (PPT) after the intervention, which were maintained over four additional weeks. Although the EBWG and VWG did not differ significantly, both outperformed the CG. In terms of self-reported pain (VAS), the VWG improved in the cervical, thoracic, and lumbar regions, while the EBWG improved their thoracic expansion (UPC, LWC) and functional capacity, indicating a positive effect of elastic resistance on mobility and function. Subjective perception of change was better in both of the intervention groups, with more sustained effects in the EBWG.

Pain assessment was conducted using two complementary approaches, in line with the definition proposed by the International Association for the Study of Pain (IASP), which defines pain as “an unpleasant sensory and emotional experience associated with actual or potential tissue damage, or described in terms of such damage” [25]. This definition highlights the need to use both subjective measures, such as the VAS (self-reported pain), and more objective ones, such as algometry (measuring pain threshold) [26].

The results showed that in the EBWG, significant differences in PPT emerged by the fourth week and were maintained over time. Notably, the changes observed in PPT in the EBWG exceeded both the MDC and MCID thresholds, supporting the clinical relevance of the improvements. Similarly, the reductions in VAS observed in the VWG surpassed the MCID for chronic non-specific pain, confirming that these differences are meaningful from a clinical standpoint rather than merely statistical. In contrast, in the VWG, improvements in VAS were also significant in the 4 weeks and persisted, but PPT improvements were not sustained in the long term. These findings reinforce the importance of using multiple methods to assess pain, as recommended in the literature [27], and confirm that incorporating elastic band exercises can enhance and prolong perceived pain thresholds over time. In contrast to observational studies that analyze pain as a statistical predictor, the present randomized controlled trial considers pain strictly as a clinical and physiological outcome, and no predictive or prognostic role is attributed to VAS or PPT within our analyses, consistent with the conceptual distinction highlighted by Figlioli et al. [28].

Both intervention groups (EBWG and VWG) showed significant differences compared to the control group, supporting the effectiveness of structured and supervised physical exercise. The improvement in pressure pain threshold in both groups could be explained by the phenomenon of exercise-induced hypoalgesia [29]. However, the EBWG showed greater sustainability of this effect, possibly due to the additional benefits of resistance training, which improves strength, muscle quality, and overall physical condition [30].

Nevertheless, as observed in previous studies such as that by Iversen et al. [31], it cannot be concluded that the observed improvements are solely attributable to the resistance component, since both groups performed vigorous walking. Therefore, it can be concluded that a structured exercise program combining aerobic activity with muscle strengthening may be effective in reducing pain in the short term. This strategy is currently recognized as a key tool in managing chronic pain in older adults, as highlighted by recent studies [32,33].

To assess pain in different anatomical regions, the visual analog scale (VAS) was chosen due to its ease of application and its usefulness in clinical settings that require broad regional evaluations. While pressure algometry (PPT) provides an objective measure of pain threshold, its application across multiple locations can be less practical in large-scale clinical environments [34]. The VAS, on the other hand, has proven to be a valid and reliable tool in studies requiring detailed regional pain assessment [35].

Regarding the results, the VWG showed a significant reduction in pain in specific areas of the back, such as the cervical, thoracic, and lumbar regions, with the latter showing sustained improvement over time. This finding may be explained by the effect of walking on subjective pain modulation, as noted by O’Connor et al. [10], who reported pain improvements following prolonged walking interventions, with more pronounced results after eight weeks. In contrast, our study achieved a moderate and statistically significant reduction in pain after just 4 weeks of supervised vigorous walking. Although the intervention period was limited to four weeks, this duration was intentionally selected to examine the onset of therapeutic effects in a relatively short and clinically feasible timeframe. The results indicate that measurable improvements in pain and function can emerge within four weeks and, importantly, that some of these benefits are maintained after cessation of supervised treatment.

The EBWG, meanwhile, showed a trend toward pain improvement, particularly in the lumbar region, with a medium effect size and sustained benefits at follow-up. This improvement may be attributed to the fact that the elastic band exercises primarily targeted the thoracic and lumbopelvic regions. However, it is possible that the 4-week intervention period was insufficient to produce more consistent subjective changes in pain, as studies such as that by Chen et al. [36] suggest that benefits may become more pronounced after eight weeks of training.

In terms of thoracic mobility, the results of this study revealed significant changes, both in the upper (UPC) and lower (LWC) measurements, after 4 weeks of intervention in the EBWG, with these changes maintained over time. The magnitude of these changes was clinically meaningful, as the improvements in UPC and LWC exceeded the established MDC for chest expansion measurements. This improvement may be attributed to the nature of elastic band exercises, which focus on postural correction, an especially relevant aspect in older adult women, who more frequently present structural spinal alterations [37]. Similar results have been reported in other studies involving older adults [38], as well as specifically in older women, such as in the study by Lee et al. [39], where elastic bands were used in both static and dynamic exercises, resulting in significant improvements in various body regions (thorax, abdomen, and lumbar area). However, those authors recommend performing the exercises at a low intensity, in contrast to our protocol, which applied high-intensity exercise based on each participant’s perceived exertion.

Although there is some controversy regarding the use of thoracic measurements via cytometry in older adults [40], subsequent studies have confirmed its reliability [16,40] and it is considered an accurate, though not highly precise, method [41]. Therefore, we can confirm that thoracic mobility improves with elastic band exercises, and this approach could also be applied to women with respiratory conditions, as combining these exercises with breathing techniques has been shown to enhance pulmonary function [42].

Regarding functional capacity, assessed using the 5XSTS, significant differences were observed in the EBWG. This improvement may be attributed to the inclusion of elastic band exercises targeting gluteal strengthening and pelvic stabilization key components in the mechanics of standing and walking. Moreover, the reductions in 5XSTS time exceeded both the MDC and MCID values reported in the literature, confirming that the functional gains observed in the EBWG are clinically significant. Previous studies have shown that elastic band exercises, even when applied in isolation, can significantly improve 5XSTS performance [39]. In the present study, the combination of these exercises with vigorous walking may have enhanced functional capacity by simultaneously improving aerobic endurance and lower limb strength, thereby facilitating a positive transfer to daily activities such as walking or rising from a chair.

Moreover, recent research has indicated that integrating elastic band exercises into multicomponent programs or combined therapies can lead to significant improvements in functional performance, including the 5XSTS, in older adult populations [43].

In terms of the subjective perception of change, both intervention groups (EBWG and VWG) showed significant differences compared to the control group. However, in the EBWG, this perceived improvement was sustained over a longer period. This may be because of the combined training, which integrates resistance exercises with elastic bands and aerobic activity (vigorous walking), potentially generating a greater sense of completeness in the intervention and, consequently, a more sustained perception of improvement in overall well-being and functionality. Previous studies have noted that multicomponent exercise programs have a greater impact on perceived improvement and treatment adherence in older adults, as they address multiple physical and functional dimensions simultaneously [44,45].

This study presents several limitations. One limitation of this study is the relatively short duration of the intervention and follow-up. However, this timeframe was intentionally selected to examine the onset and short-term maintenance of therapeutic effects. While the results demonstrate early improvements, they cannot be extrapolated to long-term chronic pain management. The generalizability of the findings is limited because the sample consisted exclusively of women aged 60 to 80 years without diagnosed cardiac or respiratory conditions and reporting non-specific musculoskeletal pain. This represents a relatively healthy, physically active subgroup of female older adults. Such selection was intentional and consistent with previous exercise-based trials, as it ensured safety and feasibility for a vigorous walking and resistance-training protocol. Nevertheless, the results may not extend to frail, sedentary, or multimorbid female older adults, and future research is needed to determine whether similar benefits are observed in these populations.

Additionally, participants’ physical activities outside the prescribed 4-week exercise program were not controlled. Although they were asked to maintain their usual routines knowing they were generally active, they were also instructed not to replicate the intervention exercises after the 4-week period, to assess whether the observed changes were sustained over time. Although no statistically significant baseline differences were found among groups for the primary outcomes, an ANCOVA-based approach could also have been used to adjust for initial variability. Future analyses may incorporate this complementary method to further refine the interpretation of group differences over time. Additionally, although the PGIC provides a global perception of improvement, it is highly influenced by expectations and the lack of participant blinding, and therefore its results should be interpreted with caution and viewed as complementary to the objective outcomes of the study.

Future research could expand the scope of the study by including women with respiratory conditions, as improvements in pulmonary function might be observed. Given the increasing life expectancy, it would also be valuable to include women over 80 years of age. Moreover, incorporating a greater variety of elastic band exercises could help determine whether more extensive protocols yield greater benefits. The results of this study should be interpreted with caution, but its strength lies in the fact that, with only three elastic band exercises combined with vigorous walking, significant improvements were achieved in a short period of time in terms of pain, chest mobility, functionality, and perceived change outcomes that are particularly relevant to the older adult population.

## 5. Conclusions

The combined intervention of elastic band exercises and vigorous walking (EBWG) proved more effective than vigorous walking alone (VWG) and the activities of the control group (CG) in improving pain threshold, thoracic mobility, functional capacity, and perceived change in older adult women with non-specific chronic musculoskeletal pain over a short intervention period. The results show that meaningful improvements can be observed after only four weeks of intervention and that some of these benefits are maintained for an additional four weeks following the end of supervised treatment. These findings highlight the clinical relevance of short-term, feasible exercise programs in older adult women, particularly in demonstrating the early onset of therapeutic effects, without implying long-term generalization beyond the timeframe studied.

## Figures and Tables

**Table 1:** Sociodemographic, anthropometric and physical characteristics of participants. Means observed, standard derivation and percentages.

Variables	Categories/Units	Total n = 102	EBWG n = 40	VWG n = 32	CG n = 30	*p*
Age (mean/SD)	Years	70.01 (5.40)	70.60 (4.92)	70.06 (5.46)	69.17 (5.99)	0.551 ^A^
Marital Status n (%)	Single	11 (10.78)	5 (12.5)	2 (6.25)	4 (13.33)	0.177 ^B^
	Married	56 (54.90)	22 (55.00)	20 (62.50)	14 (46.67)	
	Widowed	21 (20.59)	10 (25.00)	7 (21.88)	4 (13.33)	
	Divorced/separated	14 (13.73)	3 (7.50)	3 (9.38)	8 (26.67)	
Level of Education n (%)	Primary education	37 (36.27)	16 (40.00)	11 (34.38)	10 (33.33)	0.863 ^B^
	High school	14 (13.73)	6 (15.00)	5 (15.63)	3 (10.00)	
	University degree	51 (50.00)	18 (45.00)	16 (50.00)	17 (56.67)	
Weight (mean/SD)	kg	68.02 (12.52)	67.08 (12.58)	68.41 (13.28)	68.80 (11.94)	0.823 ^A^
Height (mean/SD)	m	1.60 (0.09)	1.61 (0.07)	1.61 (0.06)	1.60 (0.07)	0.908 ^A^
Body Mass Index (mean/SD)	kg/m^2^	26.64 (6.47)	26.55 (7.87)	26.35 (4.97)	27.06 (5.97)	0.909 ^A^
Charlson Index n (%)	No comorbidity	79 (77.45)	31 (77.5)	26 (81.25)	22 (73.33)	0.798 ^B^
	Low comorbidity	12 (11.76)	4 (10)	4 (12.5)	4 (13.33)	
	High comorbidity	11 (10.78)	5 12.5)	2 (6.25)	4 (13.33)	
Blood Pressure n (%)	Hypertensive	27 (26.47)	8 (20.00)	10 (31.25)	9 (30.00)	0.490 ^B^
	Normotensive	75 (73.53)	32 (80.00)	22 (68.75)	21 (70.00)	
Cholesterol n (%)	Dyslipemia	41 (40.20)	18 (45.00)	16 (50.00)	7 (23.33)	0.74 ^B^
	Normolipemia	61 (59.80)	22 (55.00)	16 (50.00)	23 (76.67)	

Note: ^A^: Anova; ^B^: Chi-square; EBWG: elastic band and walking group; VMG: vigorous walking group; CG: control group.

**Table 2:** Results of the post hoc comparisons for pain scores across groups and temporal data points (models A and B).

Variable	Group	N	T0	T1	T2
Overall VAS 0–10	EBWG	36	1.76 (1.38)	1.39 (1.51)	1.30 (1.30)
	VWG	32	2.13 (1.98)	1.19 (1.48)	1.21 (1.73)
	Within-group analyses: *p* [95% CI]; *d*		T0 vs. T1: 0.002 [0.28–1.60]; 0.53 T0 vs. T2: <0.001 [0.39–1.45]; 0.49		
	CG	23	1.53 (1.35)	1.86 (1.83)	1.52 (1.76)
PPT kg/m^2^	EBWG	36	3.63 (1.39)	3.82 (1.77)	4.29 (1.81)
	Within-group analyses: *p* [95% CI]; *d*		T0 vs. T2: 0.015 [0.09–1.21]; 0.40 T1 vs. T2: 0.019 [0.05–0.87]; 0.26		
	VWG	32	3.89 (1.53)	4.37 (1.72)	4.11 (1.85)
	Within-group analyses: *p* [95% CI]; *d*		T0 vs. T1: 0.045 [0.00–0.95]; 0.29		
	CG	23	2.94 (1.16)	2.61 (1.18)	2.73 (1.45)
	Between-groups analyses: *p* [95% CI]; *d*				
	EBWG vs. CG		-	0.020 [0.14–2.27]; 0.77	0.004 [0.41–2.69]; 0.92
	VWG vs. CG		0.042 [0.026–1.88]; 0.68	<0.001 [0.67–2.84]; 1.15	0.015 [0.20–2.54]; 0.81
VAS Head–Neck	EBWG	36	1.67 (2.29)	1.22 (2.43)	1.22 (2.04)
	VWG	32	2.19 (2.91)	1.31 (2.53)	1.25 (2.66)
	Within-group analyses: *p* [95% CI]; *d*		T0 vs. T2: 0.038 [0.04–1.83]; 0.33		
	CG	23	1.70 (2.49)	2.65 (2.85)	2.00 (2.73)
VAS Shoulder	EBWG	36	1.06 (2.28)	1.08 (2.35)	0.89 (2.08)
	VWG	32	1.50 (2.81)	0.91 (2.16)	1.19 (2.04)
	CG	23	1.87 (2.61)	1.61 (2.88)	1.39 (2.40)
VAS Dorsal	EBWG	36	0.75 (1.71)	0.97 (2.15)	0.78 (1.55)
	VWG	32	1.19 (2.17)	0.28 (0.85)	0.50 (1.34)
	Within-group analyses: *p* [95% CI]; *d*		T0 vs. T1: 0.022 [0.09–1.71]; 0.55		
	CG	23	0.17 (0.57)	0.57 (1.92)	0.74 (2.05)
VAS Lumbar	EBWG	36	3.50 (3.09)	2.36 (2.91)	2.33 (2.98)
	VWG	32	3.78 (3.23)	2.03 (2.76)	1.84 (2.84)
	Within-group analyses: *p* [95% CI]; *d*		T0 vs. T1: 0.004 [0.47–3.02]; 0.58 T0 vs. T2: 0.001 [0.69–3.18]; 0.64		
	CG	23	2.26 (3.06)	2.35 (3.43)	1.74 (3.07)
VAS Pelvis–Hip	EBWG	36	1.86 (2.93)	1.33 (2.46)	1.28 (2.22)
	VWG	32	2.03 (3.23)	1.44 (2.52)	1.28 (2.55)
	CG	23	1.70 (2.78)	2.13 (3.22)	1.74 (2.91)

Note: VAS: Visual analog scale; PPT: pressure pain threshold; EBWG: elastic band and vigorous walking group; VWG: vigorous walking group; CG: control group; T0: baseline; T1: immediately following the 4-week intervention period; T2: after a subsequent 4-week follow-up phase. Data are shown as mean (standard deviation). Results of the analyses are shown as *p* [95% confidence interval of the mean difference] and Cohen’s d (only shown when *p* is significant).

**Table 3:** Results of the post hoc comparisons for thoracic mobility and functional performance scores across groups and temporal data points (models C, D and E).

Variable	Group	N	T0	T1	T2
UPC cm	EBWG	36	3.68 (1.59)	5.12 (2.29)	4.68 (2.07)
	Within-group analyses: *p* [95% CI]; *d*	T0 vs. T1: <0.001 [−2.13–−0.74]; 0.73 T0 vs. T2: 0.003 [1.72–0.28]; 0.54			
	VWG	32	3.83 (2.21)	4.45 (2.20)	4.43 (2.01)
	CG	23	4.12 (1.47)	4.35 (2.01)	4.78 (1.91)
LWC cm	EBWG	36	3.95 (2.23)	5.48 (2.60)	5.07 (2.70)
	Within-group analyses: *p* [95% CI]; *d*	T0 vs. T1: <0.001 [−2.29–−0.76]; 0.63 T0 vs. T2: 0.003 [−1.92–−0.31]; 0.45			
	VWG	32	3.79 (1.99)	4.29 (2.64)	4.62 (2.57)
	CG	23	4.57 (2.14)	4.35 (2.34)	4.96 (2.35)
5XSST seconds	EBWG	36	8.71 (2.68)	6.87 (2.76)	7.02 (2.44)
	Within-group analyses: *p* [95% CI]; *d*	T0 vs. T1: <0.001 [0.99–2.69]; 0.67 T0 vs. T2: <0.001 [0.78–2.58]; 0.66			
	VWG	32	7.85 (2.29)	7.58 (2.31)	7.43 (2.24)
	CG	23	7.02 (2.06)	6.99 (3.15)	6.58 (3.82)
	Between-group analyses: *p* [95% CI]; *d*				
	VWG vs. CG		0.020 [0.20–3.18]; 0.34	-	-
PGIC (0–7)	EBWG	36	-	3.68 (1.41)	3.76 (1.30)
	VWG	32	-	3.25 (1.19)	3.84 (1.50)
	Within-group analyses: *p* [95% CI]; *d*	T1 vs. T2 0.008 [−1.02–−0.15]; 0.43			
	CG	23	-	4.87 (1.01)	4.65 (0.88)
	Between-group analyses: *p* [95% CI]; *d*				
	EBWG vs. CG		-	0.002 [−2.00–−0.38]; 0.93	0.032 [−1.73–−0.05]; 0.76
	VWG vs. CG		-	<0.001 [−2.45–−0.78]; 1.44	-

Note: EBWG: Elastic band and vigorous walking group; VWG: vigorous walking group; CG: control group; T0: baseline; T1: immediately following the 4-week intervention period; T2: after a subsequent 4-week follow-up phase; UPC: upper chest wall expansion; LWC: lower chest wall expansion; 5XSST: five times sit-to-stand test; PGIC: patient global impression of change. Data are shown as mean (standard deviation). Results of the analyses are shown as *p* [95% confidence interval of the mean difference] and Cohen’s d (only shown when *p* is significant).

## Data Availability

Data is available upon reasonable request.

## Supplementary Materials

The following supporting information can be downloaded at https://www.mdpi.com/article/10.3390/life16020341/s1, File S1: Elastic band protocol.

## Abbreviations

The following abbreviations are used in this manuscript:

VWG	Vigorous walking group
EBWG	Elastic band and vigorous walking group
CG	Control group
VAS	Visual analog scale
MCID	Minimal clinically important difference
PPT	Pain pressure threshold
MDC	Minimal detectable change
UPC	Upper chest expansion
LWC	Lower chest expansion
5XSTS	Five times sit-to-stand test
PGIC	Patient global impression of change
EB	Elastic bands
