# Supplementary material for: Effect of Physical Therapy with Combined Resistance Exercises and Vigorous Walking in Older Adult Women with Chronic Non-Specific Pain: A Randomized Controlled Trial
Source: Life (Basel). 2026 Feb 16;16(2):341. doi: 10.3390/life16020341 (PMC12941983; doi:10.3390/life16020341)
Supplement: Supplementary file 1 [file life-16-00341-s001.zip › life-4133747-supplementary.pdf]

File S1	
**ELASTIC BAND PROTOCOL**	
**1. Abdominal and Thoracic Breathing:**<br><br>These breathing exercises teach the patient to be aware of their breathing while performing the exercises.<br><br>Starting position: Standing with one hand on the chest and the other on the abdomen.<br><br>Perform 5 abdominal and 5 thoracic breaths.	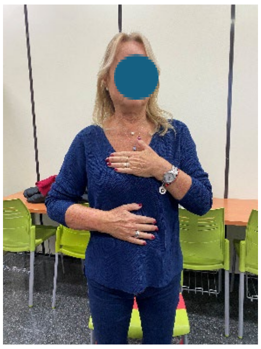
**2. Trunk extensor exercise:**<br><br>Starting position: Seated in a chair with arms relaxed.<br><br>Band placement: Sitting in the center of the band, grasp the end of the band and cross it over your back, then place the straps over your shoulders, crossing them again over your sternum. Pull the bands forward and down with tension and place them under your legs.<br><br>Step 1: Extend your torso against the resistance of the band (assume a correct position with your back straight) with slight external rotation of the upper limbs. Step 2: Gradually relax your torso, flexing it to the starting position while relaxing your arms. The return to the starting position was performed slowly by breaking the elastic band's pull.<br><br>Perform 3 sets/10 repetitions, with 1 minute of rest between sets.	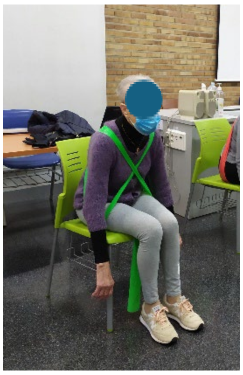 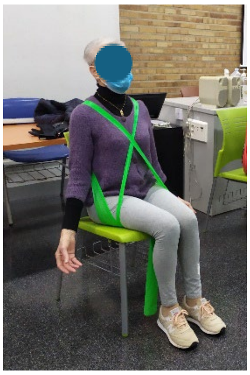
**3. Shoulder rotator cuff exercise:**<br><br>Starting position: Standing with elbows bent at 90° and wrists in a neutral position.<br><br>Band placement: Wrap the band around the back of your hand.<br><br>Step 1: Externally rotate your shoulders, maintaining the same position with your wrists. Step 2: Gradually return to the starting position. The return to the starting position was performed slowly by breaking the elastic band's pull.<br><br>Perform 3 sets/10 repetitions, with 1 minute rest between sets.	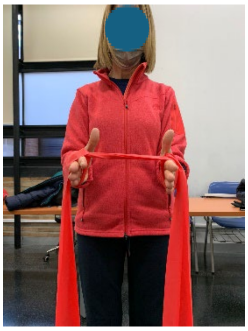 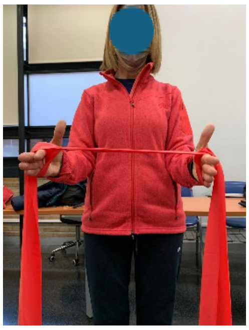

**4. Hip abductor exercise.**<br><br>Starting position: Sitting in a chair, place your heels on the floor and slightly dorsiflex your feet. Keep your knees slightly apart.<br><br>Band placement: Adjust the band around your thighs distally, near the knees, creating tension toward adduction and internal rotation.<br><br>Step 1: Separate and externally rotate your hips, separating your knees and thighs.<br>Step 2: Progressively return to the starting position. The return to the starting position was performed slowly by breaking the elastic band's pull.<br><br>Perform 3 sets/10 repetitions, with 1 minute rest between sets.	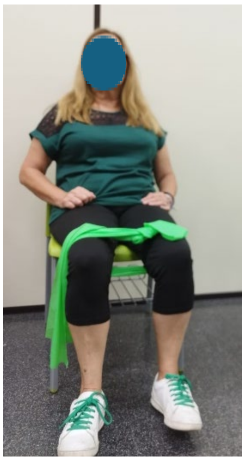 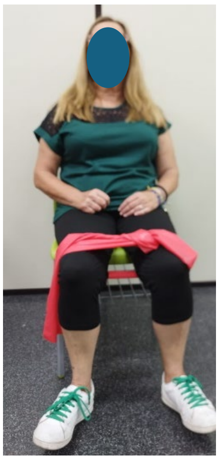
